# Gene expression in organoids: an expanding horizon

**DOI:** 10.1186/s13062-023-00360-2

**Published:** 2023-03-25

**Authors:** Artem Smirnov, Gerry Melino, Eleonora Candi

**Affiliations:** 1 Department of Experimental Medicine, Torvergata Oncoscience Research, University of Rome “Tor Vergata”, Via Montpellier 1, 00133 Rome, Italy; 2 Biochemistry Laboratory, Istituto Dermopatico Immacolata (IDI-IRCCS), 00166 Rome, Italy

**Keywords:** Organoids, Omics, Single-cell, Epigenetics, Transcriptomics

## Abstract

Recent development of human three-dimensional organoid cultures has opened new doors and opportunities ranging from modelling human development in vitro to personalised cancer therapies. These new in vitro systems are opening new horizons to the classic understanding of human development and disease. However, the complexity and heterogeneity of these models requires cutting-edge techniques to capture and trace global changes in gene expression to enable identification of key players and uncover the underlying molecular mechanisms. Rapid development of sequencing approaches made possible global transcriptome analyses and epigenetic profiling. Despite challenges in organoid culture and handling, these techniques are now being adapted to embrace organoids derived from a wide range of human tissues. Here, we review current state-of-the-art multi-omics technologies, such as single-cell transcriptomics and chromatin accessibility assays, employed to study organoids as a model for development and a platform for precision medicine.

## Background

The use of animals in biomedical research [1] and in vitro cell cultures led to milestone discoveries and the development of lifesaving treatments [2]. Nonetheless, limitations of animal models have been an Achilles heel in research of human development and disease for decades [3] as multiple processes are human-specific and therefore cannot be completely recapitulated in other animals [4]. Furthermore, conventional 2D cell cultures do not resemble the physiological tissue architecture, which limits the study of complex processes in vitro [5].

The introduction of 3D cultures derived from adult or embryonic stem cells became a breakthrough in biomedical research [6, 7], for example, for liver, pancreas [8], prostate [9] and intestinal [10] tissues. Rapid development of the isolation of adult stem cells from biopsies allowed the establishment of tissue-specific three-dimensional systems, or organoids. These achievements enable modelling of human organ development in a Petri dish including lung [11], skeletal muscle [12], bile duct [13], heart [14], neurone system [15] and hair-bearing skin [16].

**Fig. 1:**
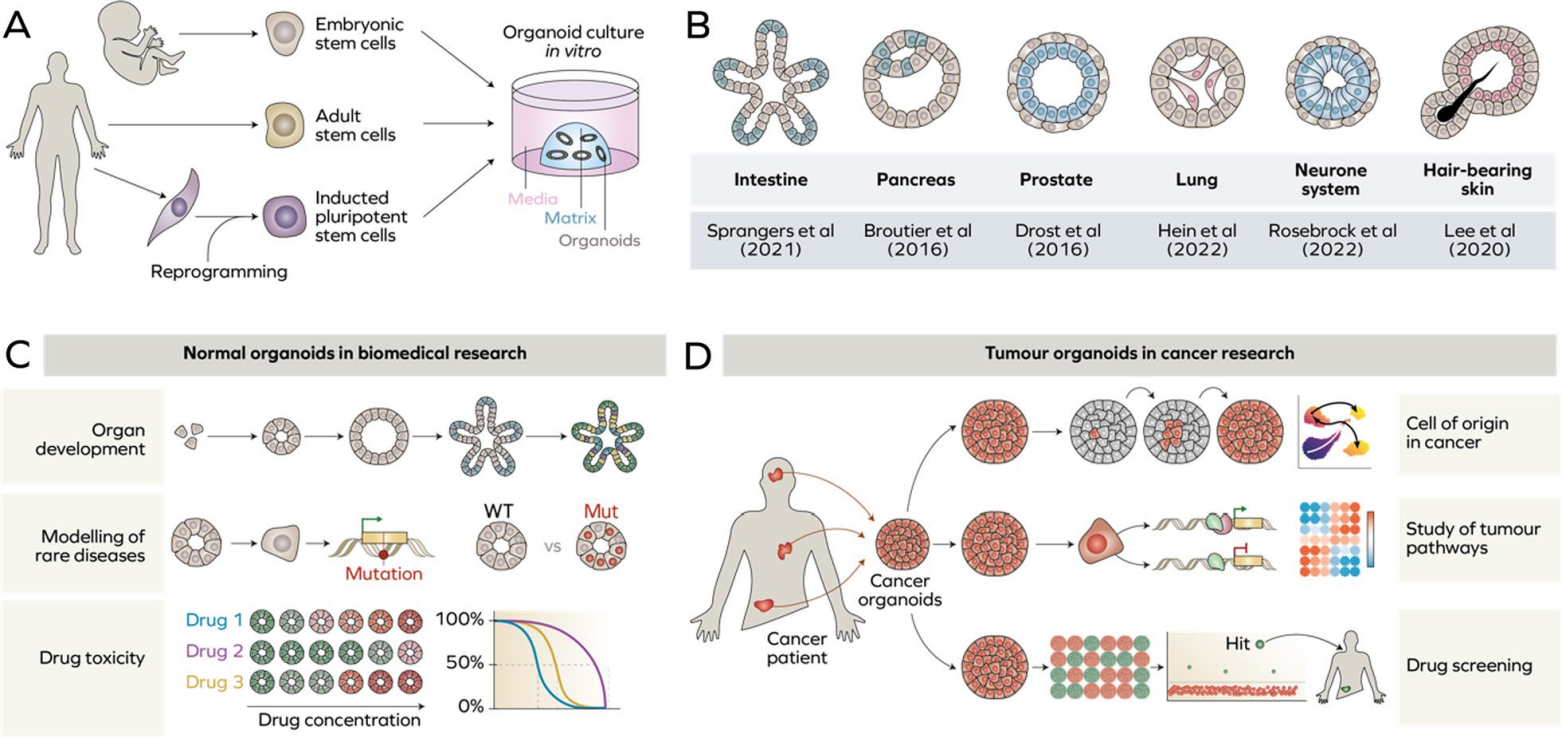
Organoids for biomedical research. **A** Organoids can be established from single embryonic, adult stem cells or reprogrammed induced pluripotent stem cells. **B** Organoid model for multiple tissues and organs have been successfully established in recent years. **C–D** Organoids are widely used in basic and applied biomedical research

Furthermore, organoids can be used to mimic biological processes, such as infection by pathogens with restricted host tropism. For instance, intestinal and gastric organoids have been proposed as a model for *Salmonella sp.* [17] and *H. pylori* [18, 19] infection, while lung organoids can be used to recapitulate *S. pneumoniae* infection of human lungs [20]. Moreover, impact of human immunodeficiency virus [21] and cytomegalovirus virus [22] on central neuronal system has been studied in cerebral organoids, while liver organoids can be used to mimic human liver infection by hepatitis B virus [23]. Remarkably, 3D cultures enabled a study of a simultaneous co-infection with distinct pathogens such as *Chlamydia* and human papilloma virus in ectocervix organoids [24]. It is noteworthy that organoids of different lineages have been extensively used during the recent COVID-19 pandemic to understand the impact of SARS-CoV-2 on respiratory airways [25], kidney [26] or eyes [27] (Fig. 1).

Multiple organoid systems rely on adult stem cells isolated from patients. More recently, the introduction of cell reprogramming [28] by using stemness factors Oct3/4 [29], Sox2 [30], c-Myc [29] or KLF4 [31] allowed the in vitro generation of induced pluripotent stem cells (iPSC) from somatic cells. iPSC soon became an attractive source of stem cells for organoid culture and enabled the generation and expansion of patient-derived organoids to study disorders that previously had no experimental models. The establishment of organoids from patient-derived iPSC opens new opportunities to study Parkinson disease [32] and epilepsy [33], heart chamber defects [34] or rare skin genetic disorders like epidermolysis bullosa [35]. Organoids based on iPSC can also serve as a platform for assessing drug toxicity, for example, in the liver [36], neurons [37] or retina [38].

Tumour organoids represent an attractive platform for personalised medicine as 3D cultures can be generated from patient-derived tumour samples, expanded in vitro and subjected to treatment with a panel of drugs in order to find an optimal therapeutic approach for a specific patient. In fact, Larsen et al. established a new pipeline for drug screening using tumour organoids [39, 40] which undoubtedly has a great potential in clinics as a platform for personalised medicine. Moreover, drug screening followed by phenotypic and multi-omics analyses provides mechanistic insights into tumour biology. For instance, recent studies employed tumour organoids to study origins of oesophageal [41], colorectal [42] and metastatic ovarian cancer [43], while other identified key epigenetic factors leading to drug resistance in colorectal [44] and breast cancer [45]. Furthermore, several studies proposed new treatment based on cancer organoids, for example targeting MEK or mTOR in colorectal cancer [46] or combined inhibition of TRAIL and CDK9 in pancreatic cancer [47].

The advances in organoid culture open the door of possibilities for mechanistical studies in very complex systems involved in multiple pathologies, such as, for example, redox balance [48–51], COVID-19 [52] or cell death [53, 54]. Therefore, employment of multi-omics poses an excellent opportunity to gain a deep understanding of physiological processes occurring in human organs [55]. Multi-omics allow global genetic, epigenetic, gene expression, and metabolic analyses. For instance, recent pancancer [56, 57] multi-omics studies have unveiled new putative targets for cancer therapy [58, 59]. In this review, we will focus on gene expression and its regulation on organoids as a research model, we will describe recent advances of sequencing techniques, and we will outline future direction in organoid research.

## Single-cell transcriptomics of organoids

Using conventional techniques to quantify gene expression, such as RT-PCR, gene array, and bulk RNA sequencing, can only provide limited information and fail to discriminate between distinct cell types present within a sample. Therefore, analysis of complex 3D cultures containing multiple cell types requires state-of-the-art technology such as single-cell transcriptomics.

**Fig. 2:**
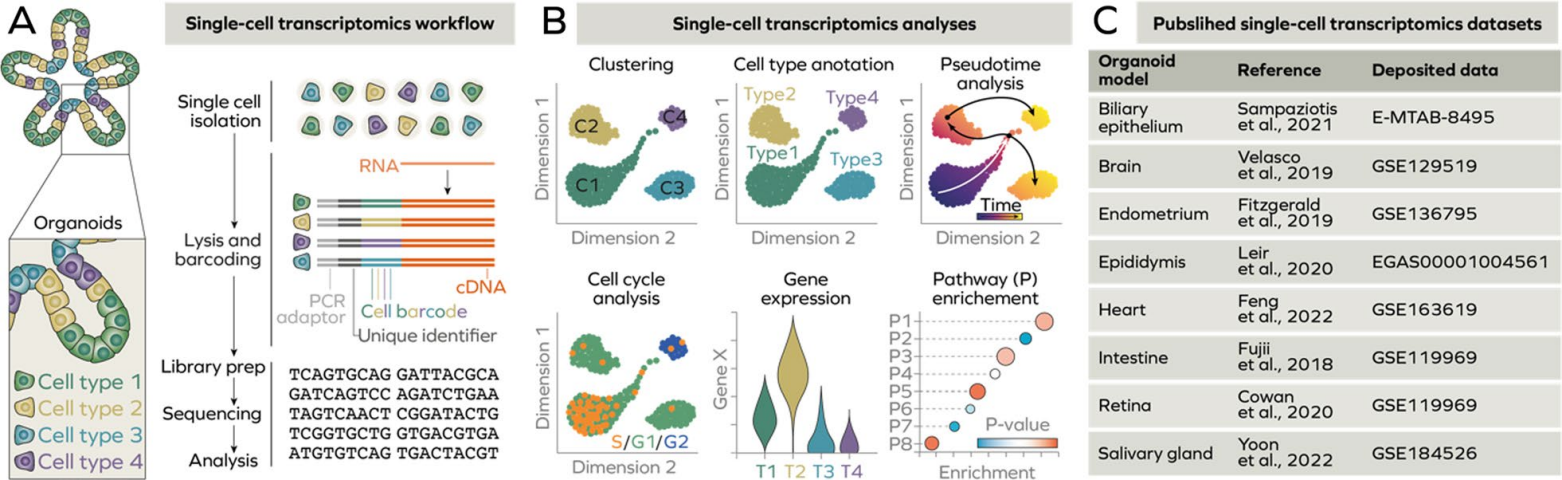
Single-cell transcriptomics of organoids. **A** Single-cell transcriptomics consists of isolation of single cells, followed by lysis, RNA conversion to cDNA by reverse transcription and barcoding. Barcoded cDNA is then used for preparation of cDNA library which can be sequenced and subsequently analysed. **B** Dimension reduction allows clustering of single cells based on transcriptome profile. Identification of highly expressed marker genes enables cell type annotation. Additional analyses can assess RNA kinetics (pseudotime analysis), cell cycle state, gene expression and pathway enrichment. **C** Recently, single-cell atlases of human organoid models corresponding different tissues have been established

Single-cell RNA sequencing (scRNA-seq) [60, 61] combines whole transcriptome amplification with next-generation sequencing at single cell level. Currently, various improved platforms have been established, allowing for the analysis of a greater number of cells at significantly lower cost. Current strategies rely on single cell isolation by cell sorting or separation in microdroplets. Then, RNA within isolated single cells is converted into cDNA and barcoded, followed by sequencing. The sequencing data are then processed, normalised, and subject to clustering, which enables identifications of cell types within the sample. Further analyses can provide information about enrichment of molecular pathways, cell cycle state, cell‐cell communication, gene expression kinetics (Fig. 2), as recently described [62]. For instance, one of the pioneering studies in the field of single-cell transcriptomics in organoids assessed distribution of cell types within mouse intestine organoids. Grün et al. identified Reg4 as a novel marker for enteroendocrine cells, a rare population of hormone-producing intestinal cells [63]. In the past seven years, scRNA-seq has been significantly improved and is now widely applied in organoid research. In this chapter, we will provide an overview of four different applications of scRNA-seq in organoid-based research.

### Comparison of organoids with matched organs and tissues

Primary advantage of 3D culture is its broader specialisation of cells dictated by three-dimensional architecture compared to conventional 2D cultures. In fact, prostate organoids derived from primary prostate cells contain additional intermediate differentiation cell types compared to the same cells grown in a Petri dish [64]. scRNA-seq allowed to compare newly established organoid models with matched liver [65], intestine [66], endometrium [67], epididymis [68], biliary epithelium [69], salivary gland [70], and heart [34] tissues (Fig. 2). Of note, transcriptome analysis at single level allows investigation of complex 3D systems, for instance, in vitro neuromuscular network [71] or bronchioalveolar lung organoids co-cultured with mesenchymal cells [72]. A series of studies underlined high level of resemblance of retinal organoids which can be successfully differentiated into key retinal cell types: retinal pigment epithelium, retinal ganglion cells, cone and rod photoreceptors, and Müller glia [73–75]. scRNA-seq has been employed to ensure high degree of similarity between tumour samples and patient-derived organoids to model gastric cancer [76] and glioblastoma [77]. Recently, a biobank of different types of paediatric kidney cancer has been established [78]. This collection, along with normal kidney organoids [79], will become a powerful tool to study kidney homeostasis and cancer in future.

### Organoids as a model for human disease

As mentioned in the introduction, organoids became an attractive platform for modelling rare diseases including genetic disorders. Importantly, correction of mutations in organoid cultures, followed by single-cell transcriptome analysis has recently showed promising results. For instance, culture of ear organoids can be used to study role of genetic alterations responsible for the hear loss [80] such as mutations in *TMPRSS3* gene [81]. Aberrant transcription of *FXN* gene causes an autosomal-recessive neurodegenerative and cardiac disorder known as Friedreich's ataxia (FRDA). Experiments aimed to rescue transcription of *FXN* gene carried out in dorsal root ganglia organoids allowed to fully reverse pathological hallmarks in vitro, as assessed by single-cell transcriptomics [82]. A series of studies focused on establishment of organoid models for neurological diseases such as autism associated with mutation in *SUV420H1*, *ARID1B*, *CHD8* [83] and *CNTNAP2* genes [84] or Prader-Willi syndrome affecting hypothalamic arcuate nucleus [85]. Furthermore, cortical organoids were used to study malformations of human cortical development caused by *EML1* gene mutations [86]. Interestingly, single-cell transcriptomics allowed to identify specific depletion of neuronal programming factors in progenitor cells within cerebral organoids derived from patients suffering from Schizophrenia [87]. Organoids bearing specific genetic alteration introduced into DNA of stem cells can be a useful tool to study initial steps of tumorigenesis [88–90], opening up new clarification on the underlying molecular mechanisms [91–95]. This approach has been adapted to model progression of colorectal cancer [96], retinoblastoma [97] as well as invasive glioblastoma [98]. Single-cell-based identification of clusters allows to detect molecular pathways behind cancerous transformation. This concept has proven useful to study tissue-specific and cell-type-specific transmission of SARS-CoV-19 infection in eyes [27], intestine [99] and kidney [100] during COVID-19 pandemic.

### Developmental studies in vitro using organoids

Refined transcriptome analyses of differentiating organoids made possible investigation of organ and tissue development in vitro*.* Recent studies provided transcriptional profiling for human embryonic liver [101], glandular epithelia [102], kidney basal membrane [103], mammary gland [104], and mesoderm [105] development. Importantly, Kim and colleagues reported establishment of embryonic body development in vitro followed by temporal single-cell analysis [106].

Development in vitro enables identification of key pathways orchestrating developmental processes. For instance, during intestinal organoids differentiation, BMP signalling controls expression of zonated genes in enterocytes [107] and Exportin 1 expression leads to an increased abundancy of Paneth cells [108], while FGF2 pathway has an essential role in salivary gland development [109]. Moreover, Motazedian et al.showed an important role for RAG1 in development of human T-cells originating from hemogenic endothelium [110]. Detailed analysis of multiple stages of retinal differentiation identified novel role of ATOH7 and Neurog2 in regulation of retinogenesis [111, 112] while temporally controlled overexpression of CCND1 led to promotion of early retinal neurogenesis [113].

Multiple studies have focused on the development of brain using 3D cultures and single-cell transcriptomics. Mouse and human cerebral organoids proved to be an excellent model for brain development in vitro [114]. 3D cultures recapitulate brain development from pluripotency, through neuroectoderm and neuroepithelial stages. Single-cell transcriptomics and pseudotime alignment allow generation of a temporal transcriptome atlas of human brain development at single-cell level [115]. For instance, this approach led to identification of three molecularly distinct subtypes of human dopamine neurons [116] as well as investigation of maturation of cerebral electrophysiologic properties [117]. Of note, inducible cell division labelling enables tracking of cell division and differentiation related pathways [118]. Furthermore, CRISPR-Cas9-based lineage tracing can be used to assess cell fate decisions during cerebral organoid development [119].

### Organoids as a platform to study tumorigenesis

As a major gene involved in tumorigenesis as well as in cancer progression, TP53 regulates distinct structures at the level of nuclear envelope [120], N6-methyladenosine methylation profile [121], reticulons [122, 123] and distinct nodes of the tumorigenic network [124–128]. Accordingly, very recent advances on the p53 biology [129–131] indicated a significant role for p53 in DNA damage response and apoptotic cell death [132, 133], ferroptosis [134], ribosome biogenesis [135] as well as ncRNA [136–139]. This complex network is clearly highly relevant for individual treatment [140] to understand the molecular mechanisms at the bases of malignant progression [141, 142] and therefore to identify specific cluster of prognostic markers [143–145], hence constituting the scientific bases for precision medicine [146–148]. In this framework, organoids play an essential role.

Lung organoids have been used to model early-stage lung adenocarcinoma [149] and to identify differentially expressed genes in alveolar epithelial progenitor cells of patients affected by smoking-associated disease [150]. Organoid model of PDAC allowed identification of new intermediate pancreatic ductal adenocarcinoma (PDAC) transcriptional cell states [151], while co-culture of PDAC organoids with endothelial cells shed light into the role of JAG1 and NOTCH pathways in cancer cell plasticity [152]. Single-cell transcriptomics of patient-derived prostate organoids unveiled presence of heterogeneous populations of prostate epithelial cells characterised by enhanced androgen signalling along with a cluster of tumour-associated club cells that may be linked to prostate carcinogenesis [153]. Analysis of colorectal cancer organoids revealed that enteroendocrine progenitor cells are enriched in *BRAF*-mutant samples and their differentiative capacities are inhibited [154]. Moreover, a cluster of stem-like cells with high expression of OLFM4 has been identified [155].

## Spatial transcriptomics of organoids

**Fig. 3:**
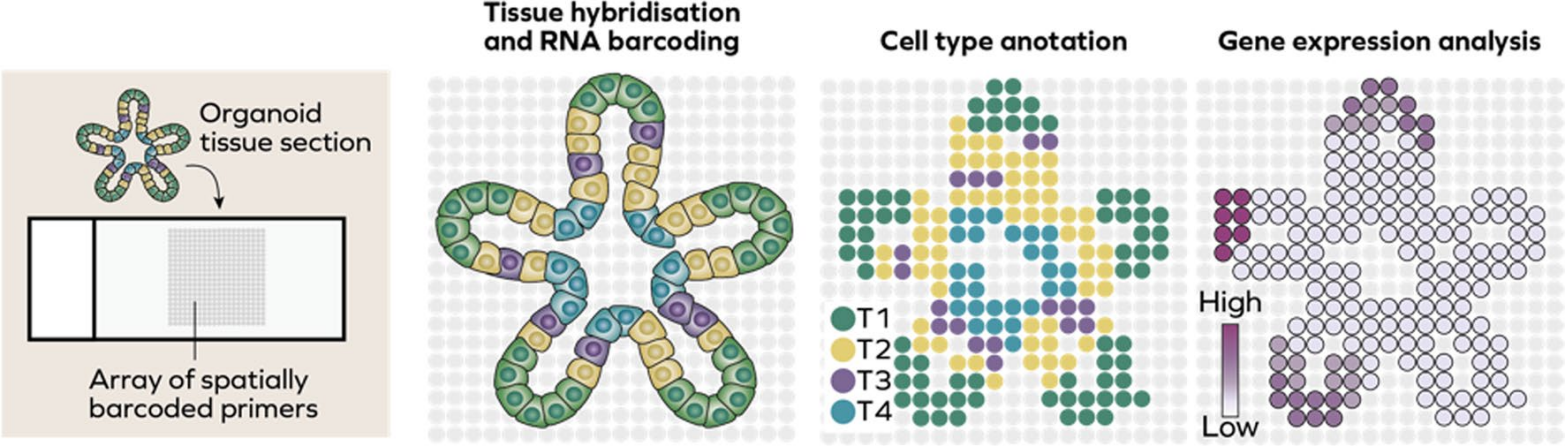
Spatial transcriptomics of organoids. In spatial transcriptomics, a tissue section is positioned on a slide covered with an array of cells containing reagents for a subsequential cell lysis and barcoding. This allows spatial reconstruction of clusters of cells followed by analysis of gene expression

A new development of single-cell multi-omics is spatial transcriptomics approach which enables analysis of gene expression in situ within a tissue sample. Technically, spatial transcriptomics can be performed in two ways. In sequencing-based techniques, the position of transcripts is labelled in situ followed by sequencing and subsequential reconstruction of the tissue map of transcription. A tissue section is placed on a slide prelabelled with RNA probes, followed by release of RNA and sequencing. Sequencing approach first reported in 2016 producing sequencing of two-dimensional sequencing map of mouse brain and human breast cancer [156]. On contrast, imaging-based approaches use the amplification of transcripts as well as sequencing directly within tissue followed by imaging. These techniques include ISH-based methods where a complementary fluorescent probe is used to label the transcript. Recent developments have introduced sequential rounds of hybridisation [157], which enable reconstruction of large tissue maps such as an atlas of mouse hypothalamic preoptic region [158] (Fig. 3).

Due to high cost of this technology, it is not widely used on organoids field. However, several recent studies showed promising results. The use of spatial and single-cell transcriptomics highlighted strong similarities between gastruloids and mammalian embryos [159]. These observations were further confirmed by tomo-seq approach which consists of the tissue embedding in cryopreservation medium and consecutive sectioning followed by RNA sequencing [160]. These data establish gastruloids as an attractive model to study development of mammalian embryos, allowing to overcome ethical limitation.

Furthermore, spatial and single-cell transcriptomics were used to assess whether endometrial organoids resemble physiological pathways in vivo. Hormone treatment of organoids resulted in creation of clusters of cells expressing secretory and ciliated populations of cells, confirming that organoids respond very similarly to the in vivo counterpart. A further pseudotime analysis revealed that organoids can be indeed used to determine cell fate decisions. These single cell transcriptomics data can be used to deconvolute bulk sequencing data from samples of endometrial tumours [161].

Of note, Fleck and colleagues developed a new platform which enables characterisation of regional composition of organoids as well as deconvolution of bulk RNA-seq of cortical organoids by using existing atlas of gene expression of developing brain, spatial expression map and accessible chromatin landscape [162]. This platform can help to integrate existing multi-omics datasets with complex organoids studies and can be used in future as a reference to establish new models.

## Organoids as a model to study regulation of expression

**Fig. 4:**
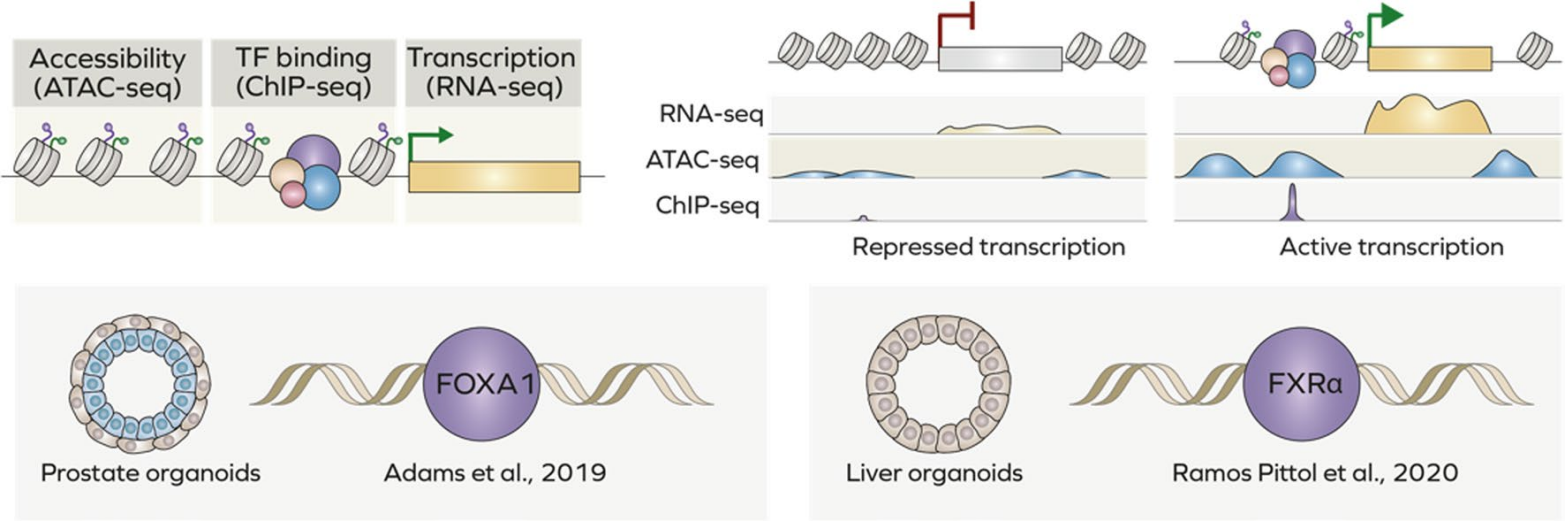
Epigenomics of organoids. (Top) RNA-seq, ChIP-seq and ATAC-seq techniques allow analysis of gene expression and its regulation at epigenetic level. Actively transcribed genes are characterised by a higher accessibility of regulatory elements (promoters and enhancers) and binding of specific transcription factors. (Bottom) Organoids have been used to study genome-wide occupancy of several transcription factors

Epigenetic modifications of chromatin are crucial for regulation of gene expression and are precisely regulated by a complex network of interaction between transcription factors, chromatin remodellers [163] and even non-coding RNAs [57, 164, 165]. Identification of genomic regulatory elements and study of epigenetic state of chromatin historically has been achieved through analyse of DNA occupancy by distinct transcription factors (TFs) and histone modifications. A chromatin immunoprecipitation followed by sequencing (ChIP-seq) assay allows identification of specific regions occupied by a transcription factor genome-wide (Fig. 4).

Despite ChIP-seq being widely used in 2D cultures, there are only a handful of studies involving organoids as it remains technically challenging [166]. For instance, mouse prostate organoids were used to unveil the role of different *FOXA1* mutations found in prostate cancer patients. Analysis of FOXA1 genome occupancy uncovered new genomic regions bound exclusively by mutant FOXA1, which alter normal program of wild-type FOXA1 and thus its role in luminal differentiation [167]. Another study comprehensively assessed the impact of distinct isoforms of the bile acid receptor (FXR) on transcription of genes involved in bile acid, fat, sugar, and amino acid metabolism in mouse liver organoids as a model. Only two isoforms, FXRα2 or FXRα4, were found to bind FXR loci, primarily by occupying ER2-motif containing regions [168] (Fig. 4).

ChIP-seq analysis of specific histone modifications allows the identification of open (active) or inactive chromatin regions genome-wide [169]. Among commonly used approaches are ChIP-seq for methylated lysine K4 and acetylated lysine K27 of histone H3 which are markers of active transcription [170]. As an alternative to mapping chromatin state by ChIP, multiple assays have been developed based on DNA accessibility for different enzymes which is correlated with chromatin state. For instance, approaches involving deoxyribonuclease (DNase) or micrococcal nuclease (MNase) have been used for long time to assess chromatin accessibility [171, 172]. DNase and MNase cleave DNA regions which are not protected by nucleosomes or occupied by TFs. The introduction of Tn5-transposase revolutionised the field. A new assay for transposase- accessible chromatin assay (ATAC) is based on identification of nucleosome-free regions which are simultaneously fragmented and labelled for further sequencing by pre-loaded transposase enzyme. The new technology allows to overcome previous obstacles, reducing the cost and requiring lower amount of material [173].

As discussed in the previous chapter, global analysis of chromatin accessibility is used to assess resemblance of organoids to original tissue at epigenetic level and thus reliability as a research model. For instance, ChIP-seq analysis of H3K4 and H3K27 methylation in mouse intestinal organoids revealed that long-term culture leads to global transcriptional changes via accumulation of H3K4me3 and loss of H3K27me3 [174]. Moreover, multi-epigenomics allow comparison of organoid culture with conventional cell cultures, such as Caco2 colorectal carcinoma cell lines. Combined RNA-seq and ATAC-seq carried out in human intestinal organoids allowed to identify transcriptional and open chromatin signatures governed by transcription factor caudal type homeobox 2 (CDX2), which are specific for organoids but not Caco2 cells grown in a Petri dish [175].

Study of chromatin accessibility in cancer organoids is becoming a new powerful tool for understanding the molecular mechanisms of tumorigenesis and development of precision medicine. For instance, using ethanol-treated colon organoids as model of alcohol-induced damage, Devall and colleagues integrated RNA-seq with ATAC-seq and identified new differentially accessibly regions of chromatin in colon organoids upon treatment with ethanol. Importantly, activation of these response factors was not found in 2D cultures, underlining the importance of three-dimensional growth conditions in recapitulating physiological environment [176]. Additionally, human colon organoids were used to study the role of vitamin D on colon homeostasis. Transcriptomics combined with ATAC-seq allowed identification of regions with increased accessibility containing VDR binding motif [177]. ATAC-seq has been proposed as a prognostic platform to profile chromatin accessibility in pancreatic cancer samples. Preliminary data revealed a subset of differentially accessible regions based on patient’s’ survival [178]. In depth analysis using ATAC-seq and H3K27ac ChIP-seq complemented by transcriptomics, led to identification of MNX1-HNF1B transcriptional axis specifically upregulated in pancreatic cancer organoids. Activation of this pathway regulated the expression of key genes responsible for maintenance of stemness of gastrointestinal cells including MYC, SOX9, and OLFM4. Moreover, high-throughput chromosome conformation capture demonstrated that expression of identified target genes was supported by specific three-dimensional chromatin architecture [179]. Interestingly, when comparing chromatin accessibility changes in colorectal cancer organoids treated with oxaliplatin, fibroblast growth factor receptor 1 (FGFR1) and oxytocin receptor (OXTR) were identified among upregulated genes, however these results were observed only in a subset of patients, highlighting complex heterogeneity of epigenetic and transcriptional response to cancer treatment [180]. Similarly, in prostate cancer organoids ATAC-seq integrated with transcriptomics allowed to identify new cancer subtypes such as stem cell–like (SCL) subtype. Interestingly, in SCL tumour cells AP-1 complex interacts with the YAP/TAZ and TEAD proteins to allow subtype-specific chromatin accessibility [181].

## Single-cell epigenomics in organoids for developmental research

Chromatin accessibility assays performed in organoids have been recently applied to developmental research. For instance, transcriptional and chromatin accessibility dynamics of human medial ganglionic eminence and generated cortex-specific organoids from human pluripotent stem allowed to confirm that proposed model can be used as a platform for generating domain-specific brain organoids to study development [182].

Furter breakthroughs are expected, as Kanton et al*.* have recently established a protocol for simultaneous RNA-seq and ATAC-seq at single-cell level in human cerebral organoids. This refined technology allows sequential transposase-based labelling of DNA and reverse transcription in isolated single nuclei [183]. Combined single-cell multi-omics can allow an in-depth analysis of cell-type specific features during organ development [184]. A recent study used combined single-cell RNA and ATAC sequencing in the developing and adult human retina and in retinal organoids derived from induced pluripotent stem cells. This comprehensive analysis revealed existence of cell type specific cis-regulatory elements (CREs) [185].

## Conclusions

**Fig. 5:**
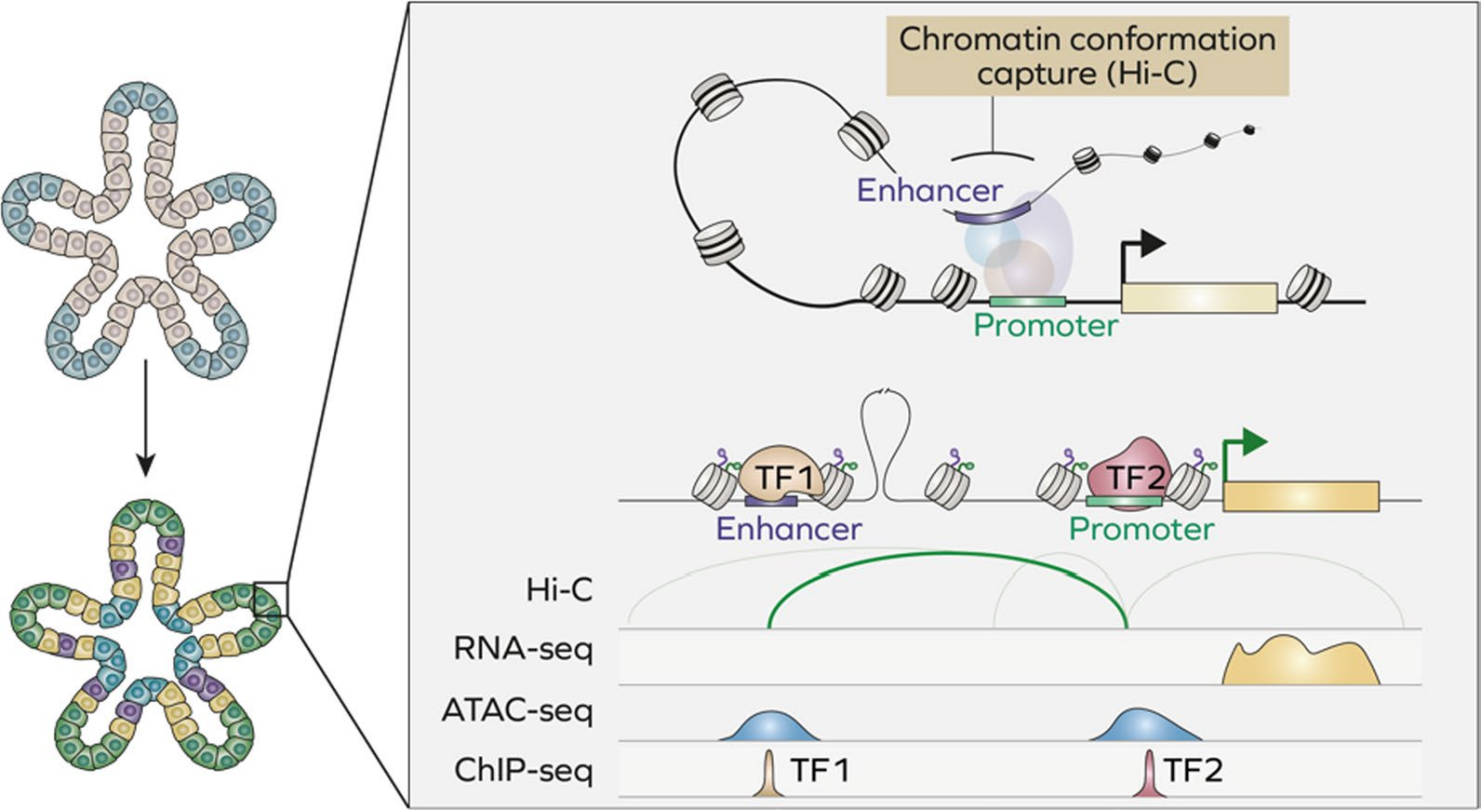
Future directions. Future developments will enable a comprehensive analysis of gene expression and chromatin state, including three-dimensional chromatin architecture, in organoids

The establishment of organoids as a research model is still in its early stages; still it is already evident their contribution to clarify the highly complex network of events leading to disease progression. As we have outlined in this review, distinct sequencing approaches have been recently employed to compare organoids with conventional cell lines and tissues. Multiple studies have successfully proved that three-dimensional cultures can in fact recapitulate tissue architecture with high degree of fidelity. These efforts have been complemented by technical optimization of omics techniques and adaptation of standard protocols to specific settings dictated by organoid growth conditions. Undoubtedly, next milestone in organoid research will be a broader, well-established application of multi-omics for basic and translational research. For instance, further improvement of spatial transcriptomics will reveal new insights into development of human organs, previously impossible due to the lack of an appropriate model. Combination of chromatin accessibility assays with chromatin conformation capture will uncover complex spatiotemporal architecture of chromatin in tissue-specific manner. Improvements of ChIP-seq technology will bring study of transcription factors to a new a level allowing to globally analyse binding profiles within virtually physiological conditions (Fig. 5). Furthermore, transcriptional analyses can be complimented by emerging single-cell proteomics to get a wider picture of gene and protein expression. Finally, application of omics to patient-derived organoids will enable prediction of treatment response for malignancies such as cancer or neurogenerative disorders, a step forward towards life-saving precision medicine.
